# Case report: A patient with HHV-6 and HHV-7 combined with *Whipple’s trophoblast* infection and *streptococcal* pneumonia

**DOI:** 10.3389/fmed.2024.1375325

**Published:** 2024-05-14

**Authors:** Heng Chen, Bo Zhao, Jing Yang, Pi-bao Li

**Affiliations:** ^1^Shandong University of Traditional Chinese Medicine, Jinan, Shandong, China; ^2^The First Rehabilitation Hospital of Shandong, Linyi, Shandong, China; ^3^Department of Pharmacy, Shandong Provincial Third Hospital, Cheeloo College of Medicine, Shandong University, Jinan, China; ^4^Department of Pharmacy, Shandong Medical College, Jinan, China

**Keywords:** Whipple’s disease, human herpesvirus, *Tropheryma whipplei*, mixed infectious pneumonia, metagenomic next generation sequencing

## Abstract

Adult respiratory distress syndrome due to viral pneumonia occurs predominantly in immunodeficient populations; adult respiratory distress syndrome secondary to human herpesvirus HHV-6 and HHV-7 pneumonia is extremely rare. Whipple’s disease, caused by *Tropheryma whipplei*, a Gram-positive bacillus and obligate intracellular pathogen, is clinically challenging to diagnose. Whipple’s disease is a chronic multisystem infectious disease caused by *T. whipplei*, most often affecting the gastrointestinal tract and joints, seldom the lungs. Both pathogens are opportunistic. We report a case of mixed infectious pneumonia in a patient with type 2 diabetes mellitus. The patient presented with dyspnea and intermittent fever. Imaging revealed multiple large patchy consolidations in the left lung. Routine anti-infective therapy was ineffective. Metagenomic next generation sequencing of bronchoalveolar lavage fluid indicated HHV-6 and HHV-7 pneumonia concurrent with *T. whipplei* and Streptococcus co-infections. Meropenem was administered to improve treatment. This case represents a rare mixed lung infection by multiple uncommon pathogens, and is of particular clinical significance.

## 1 Introduction

Human herpesviruses HHV-6 and HHV-7 are betaherpesviruses of the Roseolovirus genus, encapsulated double-stranded DNA viruses that are highly similar in terms of *in vitro* growth characteristics, genetic structure, epidemiology, and pathogenicity. HHV-6 and HHV-7 are latent persistent viruses that remain in the body after infecting humans. They can be reactivated when the immune system is suppressed (1). Currently, domestic and foreign reports on HHV-7 or HHV-6 pneumonia are scarce. *Tropheryma whipplei* (TW) is a Gram-positive bacterium and conditional pathogen that can cause the rare Whipple’s disease, primarily affecting the intestines, in immunocompromised patients. Pulmonary involvement has been reported domestically and abroad. Here we report a case of severe pneumonia in a patient with type 2 diabetes mellitus. Metagenomic sequencing of bronchoalveolar lavage fluid confirmed mixed infection by human herpesvirus (HHV) and *Tropheryma whipplei.*

## 2 Clinical data

### 2.1 Medical history

A 68-year-old male presented with a 6-day history of intermittent fever up to 39.2°C and dyspnea, and was admitted to our hospital on the night of July 26, 2023. The symptoms began 6 days prior without a clear trigger. In addition to the fever and dyspnea which worsened with activity, he experienced chest tightness, cough with white sticky sputum sometimes tinged with blood, and left-sided pleuritic chest pain during coughing episodes. He denied palpitations, headache, nasal congestion, rhinorrhea, diarrhea or abdominal pain.

Past medical history was significant for a 4-year history of diabetes mellitus, for which he was taking gliclazide regularly and felt his blood glucose was fairly well controlled.

On admission, vital signs were: T 38.3°C, P 90 bpm, R 23/min, BP 129/75 mmHg. He appeared acutely ill and was breathing with difficulty, but was mentally clear though in poor spirits and uncooperative with examination. Pupils were equal, round and reactive to light. Cyanotic lips. No pharyngeal congestion. Coarse breath sounds on the right lung, decreased breath sounds on the left lower lung, and extensive fine moist rales bilaterally over the lung bases.

### 2.2 Auxiliary examinations (Table 1)

Patient laboratory test results and partial clinical data shown in Table 1.

**Table 1:** Patient laboratory test results and partial clinical data.

Patient characteristics	Admission (2023-07-26)	2023-08-04	Reference range (Units)
Gender	Male	—	—
Age	68 years old	—	—
NT-ProBNP	1468.00↑	56.00	0–900 (pg/mL)
WBC	12.72↑	6.62	3.5–9.5 (10^9/L)
NEU%	87.7%↑	66.8	40–75%
LYM%:	6.0↓	25.6	20–50%
HGB	118.0 ↓	121.0 ↓	130–175 g/L
PLT	106.0 ↓	261	125–350 (10^9/L)
bCRP	149.85↑	9.54↑	0–5 mg/L
SAA	>200.00↑	>43.97↑	0–10.0 mg/L
PCT	0.89 ↑	0.14 ↑	0–0.05 ng/mL
IL-6	2,255↑	4.69	0–10 pg./mL
PT	14.40s↑	—	10–14 s
APTT	45.50s↑	—	22–38 s
TT	12.40s↓	13.80s↓	14–21 s
FIB	7.08 ↑	4.28 ↑	2–4 g/L
AST/ALT	0.51↓	0.41↓	0.75–1.25
Cholinesterase (CHE)	3705.9↓	4501.6↓	4,620–11,500 U/L
Total bile acid (TBA)	13.8↑	1.9	0–10umol/L
Total bilirubin (TBIL)	28.50↑	11.10	0–21umol/L
Direct bilirubin	10.6↑	2.7	0–6.8umol/L
Albumin	32↓	31.9↓	35–55 g/L
(Uric acid) UA	156.1↓	214.8	210-430umol/L
PO_2_ (4 L/min O_2_)	60.78↓	—	80-100 mmHg
Calcium Ca	1.99↓	1.99↓	2.08–2.6 mmol/L
Glycated hemoglobin (HbAIC)	6.6↑	—	4–6%
D-Dimer	—	3.85↑	0–1 ug/mL
LDH	187	—	72–182 U/L
Oxygen saturation	90%↓		95–100%
PCO_2_ (4 L/min O_2_)	31.31↓	—	35–45 mmHg
HIVAb	(—)		
HBSAg	(—)		
anti-HCV	(—)		
anti-TP	(—)		

### 2.3 Imaging findings

**Figure 1:**
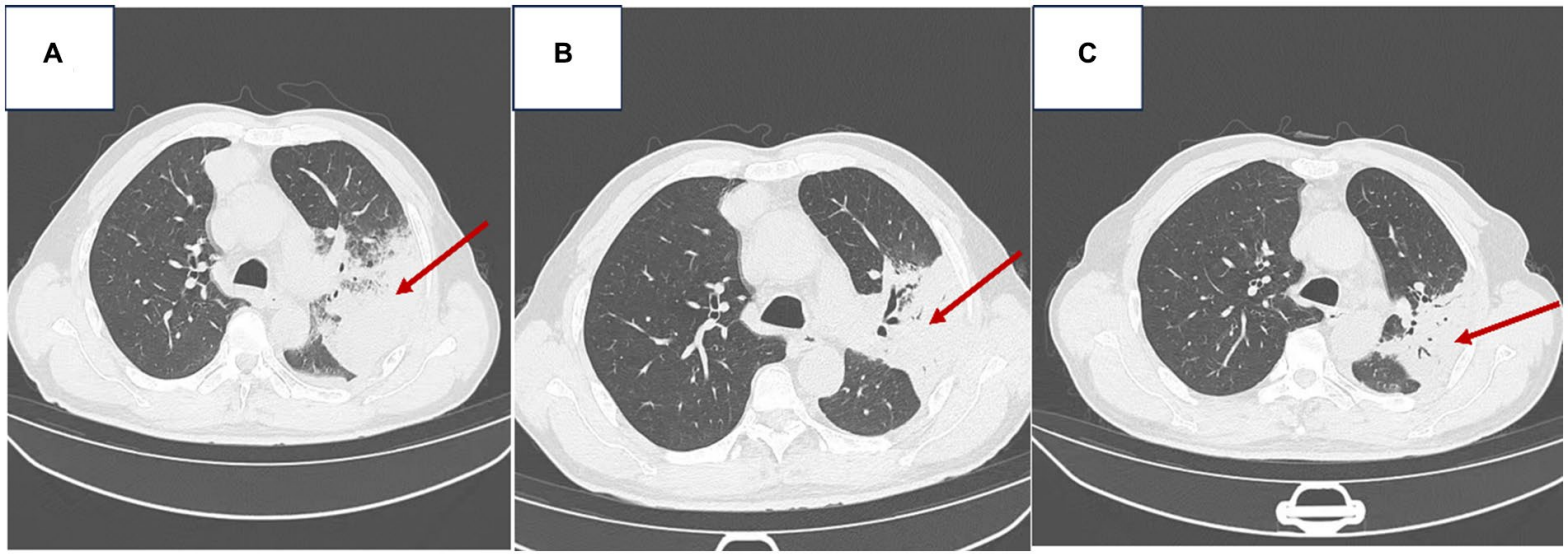
Chest imaging of the patient [**(A)** Admission chest CT on 07/26/2023; **(B)** Follow-up chest CT on 08/03/2023; **(C)** Follow-up chest CT on 08/10/2023].

Serial chest CT scans were performed on admission (day 0, 07/26/2023) and after treatment on days +8 to +15. The admission CT demonstrated (1) left lobar pneumonia with multiple large patchy consolidations throughout the left lung, prompting recommendation for short-term follow-up. (2) A right paravertebral soft tissue density mass along the anterior superior mediastinum measuring 58 mm x 55 mm x 72 mm with clear, irregular margins, prompting recommendation for contrast-enhanced CT. (3) Small bilateral pleural effusions and pulmonary atelectasis. (4) Mild bilateral pulmonary emphysema (Figure 1A).

The follow-up CT on day +8 (Figure 1B) showed decreased patchy consolidations in the left lung compared to admission, otherwise no change. The CT on day +15 (Figure 1C) demonstrated further decrease in the left lung consolidations compared to prior scans. Findings were consistent with interval improvement of left lobar pneumonia and decreasing left pleural effusion.

Echocardiography and electrocardiography showed no significant abnormalities.

Admission diagnoses: (1) Community-acquired pneumonia (non-severe); (2) Type 2 diabetes mellitus; (3) Mediastinal mass.

### 2.4 mNGS sequencing

mNGS results of patient’s bronchoalveolar lavage fluid shown in Table 2.

**Table 2:** mNGS results of patient’s bronchoalveolar lavage fluid.

Bacteria/Viruses	Genus	Species	Read count	Pathogen concentration (copies/mL)
G+	Tropheryma	*Tropheryma whipplei*	1,364	<1e+2
G+	Streptococcus	*Streptococcus*	133	<1e+2
DNA virus	Roseolovirus	Human herpesvirus 7	1,392	<1e+2
DNA virus	Roseolovirus	Human herpesvirus 6	1,211	<1e+2

## 3 Treatment course

Upon admission, the patient was placed under cardiac monitoring and received high-flow oxygen therapy (4 L/min). Treatment included aminophylline for bronchial asthma, carboxymethyl cellulose for cough relief, nebulization, gastric protection, blood sugar control, correction of electrolyte imbalances, and supportive care. Empirical antibiotic therapy with “Piperacillin-Tazobactam + Levofloxacin” was initiated; however, despite treatment, significant amounts of yellowish-white purulent sputum continued to be expectorated, accompanied by intermittent fever ranging between 37.0°C to 38.3°C. To identify pathogens, fiberoptic bronchoscopy was performed, and bronchoalveolar lavage fluid underwent high-throughput DNA sequencing. On the second day of admission, *Tropheryma whipplei*, *Streptococcus*, Human Herpesvirus 7, and Human Herpesvirus 6 were identified (refer to Table 2). No fungi, parasites, or atypical pathogens were detected. Consequently, the antibiotic regimen was adjusted to “Meropenem 0.5 g IV drip q8h.” Subsequently, the patient’s fever markedly subsided. Within the first week, there was no recurrence of fever, marked improvement in mental status, reduced sputum production, disappearance of lung crackles, and a notable decline in inflammatory markers such as blood cell count, procalcitonin, IL-6, among others. Bronchoscopic findings in the left lung of the patient: The left lower lobe showed abundant rust-colored foamy sputum filling; the tracheal mucosa showed edema and scattered hemorrhage.

On the 7th day of admission (2023-08-03), a chest CT scan revealed the following findings: 1. Improvement in left lung lobar pneumonia compared to previous imaging. 2. Minimal changes in a soft tissue density mass at the right margin of the anterior mediastinum, prompting a recommendation for enhanced CT examination. 3. Presence of an air-containing cavity in the right lower lung lobe requiring periodic follow-up. 4. Resolution of right pleural effusion and reduced left pleural effusion; bilateral lung collapse was alleviated (refer to Figure 1B). The patient continued to receive anti-infective and symptomatic supportive therapy. Pathological results of bronchoscopy in the left lung of the patient: Lymphocytic and neutrophilic infiltration seen, interstitial congestion and edema, focal squamous metaplasia of mucosal epithelium.

A subsequent chest CT on 2023-08-10 revealed: 1. Improvement in previously observed left lung lobar pneumonia, along with reduced left pleural effusion. 2. Minimal changes in the soft tissue density mass at the right margin of the anterior mediastinum compared to previous scans, warranting enhanced CT examination. 3. No changes in the air-containing cavity in the right lower lung lobe (compared to previous imaging) (refer to Figure 1C). A mediastinal MRI scan, both plain and enhanced (using a GE 1.5 T device), showed enhanced abnormal growth in the middle anterior mediastinum, suggestive of a potentially invasive thymoma. Further investigations via PET-CT and tissue pathology examination were recommended. By 2023-08-11, the patient’s condition had improved, showing signs of recovery by the 16th day of admission, along with a recommendation for post-discharge surgical consultation.

## 4 Discharge diagnosis

Severe pneumonia caused by HHV-6, HHV-7, *Whipple’s trophoblast* and *Streptococcus* infection.

Type 1 respiratory failure. Mediastinal mass. Type 2 diabetes.

## 5 Discussion

To date, eight human herpesviruses (HHV) have been shown to infect humans. Human herpesvirus 6 (HHV-6) was first isolated from AIDS and lymphoproliferative disease patients in 1986, and human herpesvirus 7 (HHV-7) was isolated from healthy peripheral blood mononuclear cells (PBMCs) in 1990 (2). HHV-7 and HHV-6 infections are very common in the population. Primary infections most commonly occur in infants and young children, often with good prognosis. After primary infection, HHV-7 and HHV-6 usually persist long-term in a latent form within the host. HHV-7 and HHV-6 can be reactivated in states of immunosuppression or immunodeficiency in the host, leading to symptomatic infections (3). Amanati et al. (4) performed bronchoalveolar lavage on 83 critically ill ventilated children and estimated prevalence of HSV-1, HHV-6, HHV7, EBV, and HCMV as approximately 2.4, 13.2, 2.4, 7.2, and 2.4%, respectively, by PCR.

In the human body, HHV-6 infects tissues of the central nervous system (CNS), tonsils, salivary glands, kidneys, liver, lymph nodes, endothelial cells, and mononuclear/macrophage cells; while HHV-7 can be detected in lymphoid tissue, salivary glands, tonsils, liver, kidneys, lungs, and skin. Little is known so far about the infective process within the host body after viral entry via blood or respiratory routes. Additionally, mononuclear macrophages and CD4+ T lymphocytes are thought to be potential sites of latent infection for HHV-6 and HHV-7, respectively (3).

Reactivation of latent viruses in the host may be associated with infective and immunological factors. Activation of HHV-7 and HHV-6 has been associated with diseases of the CNS, bone marrow, lungs, gastrointestinal tract, and liver (3). It is commonly reactivated in AIDS patients, hematopoietic stem cell transplant (HSCT) or solid organ transplant (SOT) recipients, and people with immunodeficiency (e.g., those on radiotherapy, chemotherapy, immunosuppressants), where HHV-6 and HHV-7 activation exacerbates disease progression. Symptoms include fever, rash, hepatitis, pneumonia, and myocarditis (5).

With advances in next-generation pathogen diagnostic technology, metagenomic NGS (mNGS) has significant advantages in diagnosing rare bacterial, viral, and mixed infections. There have been sporadic reports of pulmonary infections caused by *Tropheryma whipplei*. Whipple’s disease (WD) is a rare multisystemic destructive illness caused by infection with *Tropheryma whipplei* (TW), a Gram-positive bacterium. The incidence is estimated around 1 in 1,000,000 (6). In immunocompromised patients, it can lead to the rare Whipple’s disease, primarily affecting the intestines (main manifestations of abdominal pain, malabsorption diarrhea, or weight loss), while pulmonary involvement is very rare. Patients with pulmonary infection may present with nonspecific symptoms like fever, chronic cough, dyspnea, and wasting (7). Currently, the pathogenic mechanisms of TW-induced pneumonia remain unclear, and treatment regimens for TW pneumonia are also incomplete (8).

One study summarized the most common chest imaging findings in 20 cases of *T. whipplei* pneumonia as nodules (50%), which could be isolated or diffuse, with ground-glass or solid components, ranging from small to several centimeters in size. This was followed by interstitial changes (25%) and patchy infiltrates (25%). Enlarged hilar and mediastinal lymph nodes were seen in 4 cases (20%) (9). In our case, the main chest CT findings were multiple large patchy consolidations in the left lung, accompanied by a right paravertebral soft tissue density mass along the anterior superior mediastinum, and small bilateral pleural effusions. Compared to published cases of HHV6/HHV7 pneumonia and chest CT/radiographs of TW pneumonia, along with this patient’s pulmonary imaging features, the CT findings were relatively distinct, showing large multi-focal high density patches unilaterally (left lung), while the contralateral lung was almost free of significant pathology, differing clearly from the solitary or diffuse, ground-glass nodules in 50% of TW pneumonia cases, and also differing from reported CT changes in HHV6/HHV7 pneumonia. It is reported that 30–40% of typical WD patients have pulmonary involvement, manifesting as pleural effusions, pulmonary infiltrates, granulomatous mediastinal adenopathy, or mediastinal lesions (10). In our patient, MRI of the mediastinum with and without contrast demonstrated an intensely enhancing mass in the anterior and middle mediastinum, considered an invasive thymoma. This may further add to our diagnosis. Unfortunately, the patient could not be convinced to undergo further pathologic diagnosis.

The most common gastrointestinal manifestation of Whipple’s disease is intestinal malabsorption of fat. Upper endoscopy mainly shows histologic injury to the duodenum and small intestine mucosa, villous hyperplasia and deformity, and macrophage PAS stain positivity on biopsy. *Tropheryma whipplei* can also be detected on electron microscopy. Additionally, diagnosis can be established by typical histologic changes in visceral pathology, together with positive pathogen findings (11). Currently, the pathogenic mechanisms of TW-induced pneumonia remain unclear, and treatment regimens for TW pneumonia are also immature. More common treatments for Whipple’s disease are intravenous ceftriaxone (2 g daily) or meropenem (1 g three times daily) along with trimethoprim/sulfamethoxazole (160/800 mg twice daily) for over 1 year. Clinical symptoms gradually improve over 7–21 days, but approximately 20% of patients may experience relapse. The mechanisms of relapse remain unclear. Studies have found *T. whipplei* to have inherent resistance to trimethoprim-sulfamethoxazole, with alternative agents being doxycycline (200 mg/day) and hydroxychloroquine (200 mg three times daily) (12).

Some studies have confirmed co-infections of *T. whipplei* with other pathogens in the lungs (13). However, there are currently no reported cases of co-infection with HHV6 or HHV7 in pulmonary infections. We collected reported cases of HHV6 and HHV7 pneumonia over the past few years (Table 3), and found most patients presented with dyspnea at onset, which was also the situation in our reported mixed infection case. Previously, we tended to think of HHV6 and HHV7 viruses along with *T. whipplei* as opportunistic dormant infections that could be activated by the patient’s immunosuppression or other factors, leading to disease. However, there have been reports of young patients without underlying illness or immunodeficiency. We suspect activation of HHV6 and HHV7 viruses, including *T. whipplei*, may actually result from tissue injury caused by underlying pulmonary pathology, initiating a cascade leading to pathogen activation.

**Table 3:** Reported cases of HHV-6 and HHV-7 pneumonia.

Herpesvirus type	Gender	Age	Clinical symptom	Diagnosis and outcome	Comorbidities	Diagnostic method	Imaging
HHV-6 (15)	F	19 years	Dyspnea, cough, fever, nausea	Pneumonia, ARDS with respiratory failure leading to death	None	HHV6-DNA detected by PCR in lung biopsy	CXR showed diffuse bilateral infiltrates
HHV-6 (16)	F	45 years	Dyspnea, dry cough, headache	HHV-6 viremia, pneumonia, and meningoencephalitis, recovered	None	HHV-6 detected by PCR in BAL, CSF, serum	CT showed extensive bilateral airspace disease & ground glass opacities
HHV-7 (17)	F	71 years	Fever, cough, dyspnea, chest pain	Pneumonia, ARDS with type 1 respiratory failure, recovered	None	Viral load in BALF, lung biopsy	CT showed extensive consolidative & ground glass opacities, nodular opacities
HHV-7 (18)	F	46 years	Cough, sputum, fever, mild dyspnea	CAP, HHV-7 infection, recovered	Viral hepatitis, hypothyroidism, hyperglycemia	BAL mNGS	CT showed bilateral patchy densities, nodules with halo sign, progressive cavities

Our patient was an elderly male, with acute onset mainly respiratory symptoms, multiple comorbidities, and longstanding poor glycemic control. High blood glucose levels can lead to reduced macrophage function (14), impaired population immunity, which may be high risk factors for *T. whipplei* infection. Due to the low incidence and low clinical specificity of *T. whipplei* and human herpesvirus pneumonia, they are often misdiagnosed or have delayed treatment. With advances in molecular biological diagnostic techniques, especially application of BALF-mNGS testing, early diagnosis of TW and human herpesviral pneumonias has greatly improved, reducing antibiotic misuse and positively impacting patient outcomes.

## Data availability statement

The original contributions presented in the study are included in the article/Supplementary material, further inquiries can be directed to the corresponding authors.

## Ethics statement

The studies involving humans were approved by Ethics Committee of Shandong First Rehabilitation Hospital. The studies were conducted in accordance with the local legislation and institutional requirements. The participants provided their written informed consent to participate in this study. Written informed consent was obtained from the individual(s) for the publication of any potentially identifiable images or data included in this article.

## Author contributions

HC: Writing – original draft, Writing – review & editing. BZ: Supervision, Writing – review & editing. JY: Funding acquisition, Writing – review & editing. P-bL: Supervision, Writing – review & editing.
