# Supplementary figures and images for: Case report: A patient with HHV-6 and HHV-7 combined with Whipple’s trophoblast infection and streptococcal pneumonia
Source: Front Med (Lausanne). 2024 May 14;11:1375325. doi: 10.3389/fmed.2024.1375325 (PMC11130432; doi:10.3389/fmed.2024.1375325)

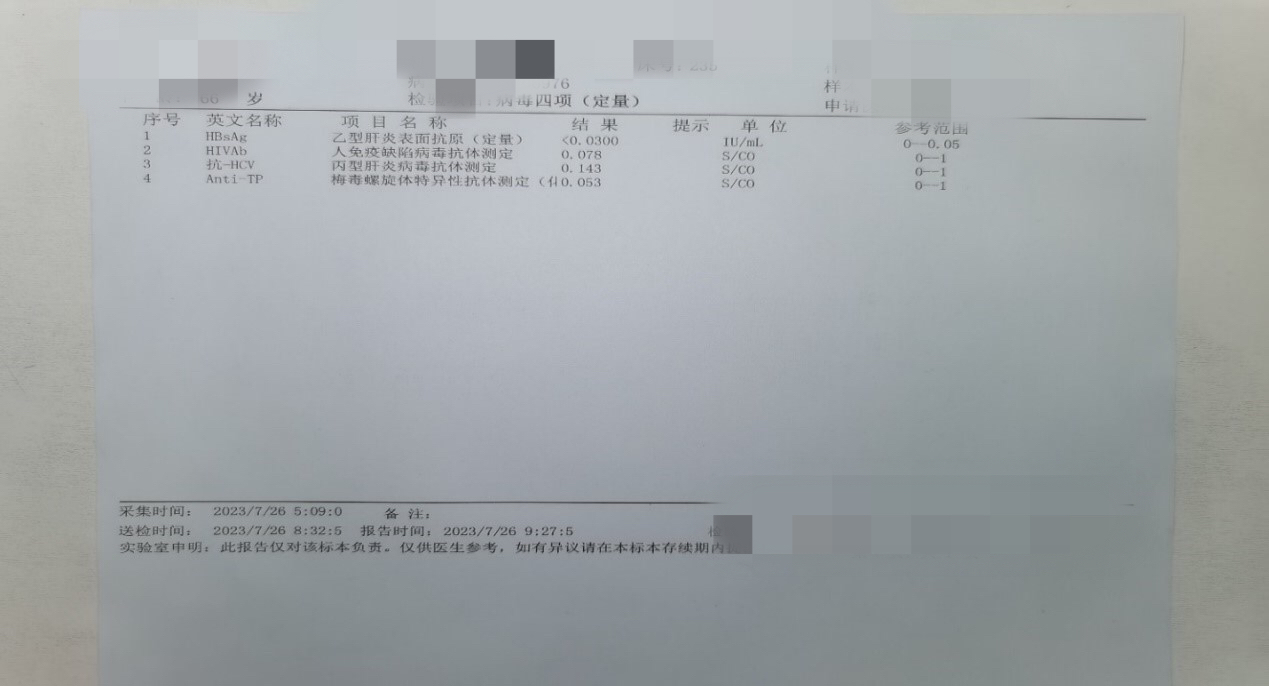

Supplement: Supplementary file 1 [file Image_1.jpg]
